# Low-Intensity Blast Exposure Induces Multifaceted Long-Lasting Anxiety-Related Behaviors in Mice

**DOI:** 10.1089/neur.2024.0134

**Published:** 2024-12-12

**Authors:** Heather R. Siedhoff, Shanyan Chen, Ashley Balderrama, Dejun Jackson, Runting Li, Grace Y. Sun, Ralph G. DePalma, Jiankun Cui, Zezong Gu

**Affiliations:** ^1^ Truman VA Hospital Research Service, Columbia, Missouri, USA.; ^2^ Department of Pathology & Anatomical Sciences, University of Missouri School of Medicine, Columbia, Missouri, USA.; ^3^ Office of Research and Development, Department of Veterans Affairs, Washington, District of Columbia, USA.; ^4^ Department of Surgery Uniformed Services, University of the Health Sciences, Bethesda, Maryland, USA.

**Keywords:** anxiety-like behaviors, open-field blast, primary blast brain injury, sociability

## Abstract

Primary blast exposure is a predominant cause of mild traumatic brain injury (mTBI) among veterans and active-duty military personnel, and affected individuals may develop long-lasting behavioral disturbances that interfere with quality of life. Our prior research with the “Missouri Blast” model demonstrated behavioral changes relevant to deficits in cognitive and affective domains after exposure to low-intensity blast (LIB). In this study, behavioral evaluations were extended to 3 months post-LIB injury using multifaceted conventional and advanced behavioral paradigms. C57BL/6J male mice, aged 2 months old, were subjected to a non-inertial primary LIB-induced mTBI by detonating 350 g of C-4 at a 3-m distance on 1-m-tall platforms. Three months after injury, mice were evaluated using the open-field test (OFT), social interaction test, and advanced Erasmus Ladder paradigm. With OFT, no apparent anxiety-like changes were detected with the LIB-exposed mice and sham controls, and both groups displayed similar center-zone activities. Although no social interaction parameters reached significance, a majority of LIB-exposed mice *initiated* less than 50% of interactions compared with their interaction partners, suggesting decreased sociability. With the Erasmus Ladder test to assess motor functions, associative learning, and stimulus response, LIB-exposed mice appeared to display increased instances of leaving before the cue, reminiscent of “escape behavior,” indicative of anxiety-related activity different from that OFT detected. Overall, these results revealed subtle multifaceted long-lasting anxiety-relevant effects following LIB exposure. The “Missouri Blast” platform offers a basis for future research to investigate the underlying biological mechanism(s) leading to domain-specific behavioral changes.

## Introduction 

Mild traumatic brain injury (mTBI) is the predominantly reported severity in military contexts^1^ and frequently caused by low-intensity blast (LIB) exposure.^2,3^ High-order explosives can generate blast waves that undergo kinetic energy transfer^4^ upon contacting brain tissue, and LIB exposure can damage subcellular components.^5–7^ This type of “invisible” injury of structural brain abnormalities is non-detectable by conventional neuroimaging techniques.^2^ Nevertheless, although affected subjects can be quickly transferred from the battlefield for recovery, they may face the risk of developing physical and mental disturbances beyond the expected recovery period, often impeding work efficiency and degrading the quality of life.^8^

Clinical researchers are becoming increasingly aware of the relationship between blast exposure and aberrant neurobehavioral outcomes, such as anxiety,^9,10^ impaired psychosocial behaviors,^11,12^ cognitive deficits,^13–16^ and motor functions.^17,18^ Yet, the trajectory of these consequences requires careful elucidation. To this end, pre-clinical research has shown aberrant behavioral outcomes related to anxiety and cognitive deficits after blast exposures at various intensity levels and time points.^6,19–21^ Previous studies using the “Missouri Blast” mouse model also revealed acute-to-subacute phase behavioral deficits after LIB exposure.^6^

More recently, using a sensitive home-cage monitoring system, we observed changes in aversive light-induced anxiety-like behaviors in mice 3 months after LIB exposure.^22^ In addition, using the home-cage with the CognitionWall assessment, we observed deficits in discrimination learning and cognitive flexibility in LIB mice at 3 months post-injury.^23^ These results motivated us to further examine multifaceted behavioral activities using more advanced and automated paradigms after long-term LIB injury.

In the present study, we examined behavioral deficits using paradigms equipped with the EthoVision software for single-animal tracking in the open-field arena and multi-animal tracking during social interaction. Mice were then subjected to the Erasmus Ladder test to investigate motor functions, associative learning, and stimulus response over the course of 8 days. Overall, this study incorporated a blast model and multifaceted behavioral assessments that helped to delineate whether single LIB exposure could lead to long-lasting behavioral consequences.

## Materials and Methods

### Animals

All experiments were performed in a double-blinded manner and in accordance with the University of Missouri-approved protocols for the Care and Use of Laboratory Animals and the Animal Research: Reporting of In Vivo Experiments (ARRIVE) guidelines. Forty C57BL/6J male mice (RRID:IMSR_JAX:000664; The Jackson Laboratory, Bar Harbor, ME) aged 2 months old were housed in groups with a 12-h light/dark cycle (lights on/off at 7:00 am/7:00 pm) in standard mouse cages containing bedding and provided food and water *ad libitum*. Body weights were obtained prior to blast exposure and sacrifice.

### Open-field LIB setting

**FIG. 1.**
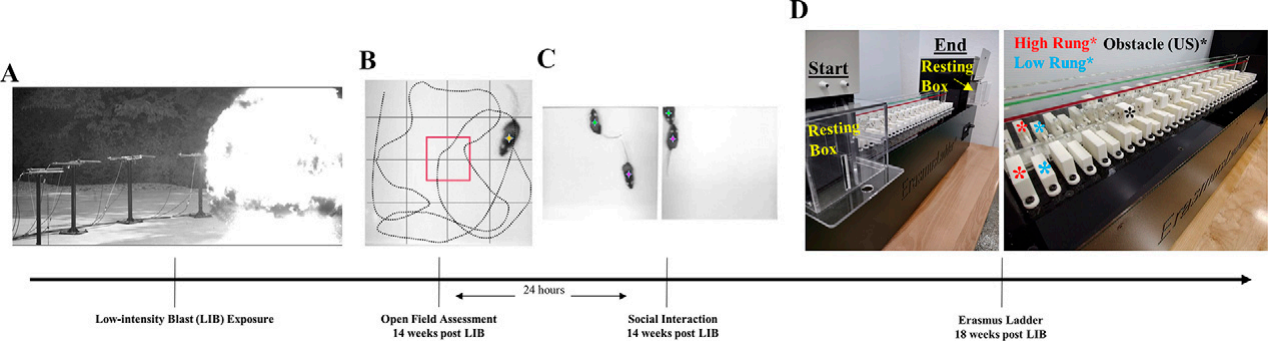
Experimental setup and timeline. LIB exposure was carried out in an open-field environment with mice placed in the prone position on platforms distanced 3 m away from C4 detonation **(A)**. Open-field behavioral assessment was performed 14 weeks post-LIB exposure **(B)** and social interaction behavioral assessment 24 h later in the same environment **(C)**. Erasmus Ladder behavioral assessment was performed 18 weeks post-injury, involving 4 days of training (sessions 1–4) and 4 days of challenge sessions (sessions 5–8) **(D)**. LIB, low-intensity blast.

Open-field LIB exposure was conducted at the Experimental Mine open-field blast quarry at the Missouri University of Science and Technology.^5,6,24,25^ Characterizations of LIB exposure and environmental conditions have been detailed elsewhere.^6^ Briefly, mice were assigned randomly to two groups: LIB-exposed mice (*N* = 20) and sham controls (*N* = 20). Mice were anesthetized with an intraperitoneal injection of 10 μL/g body weight of ketamine/xylazine mixture (12.5 mg/mL ketamine and 0.625 mg/mL xylazine) and transported to the blast field within 30 min. Mice in the sham control group underwent identical anesthesia procedures but without LIB exposure. Mice were placed in the prone position in metal-mesh holders on a platform 1-m above ground and 3 m away from the detonation of a 350-g sphered C-4 generating a magnitude of 46.6 kPa peak overpressure and a maximum impulse of 60.0 kPa*ms (Fig. 1A).^5,6,24–26^ Mice did not show head or bodily motion during the blast. They were monitored until recovery from anesthesia and allowed access to food and water *ad libitum*.

### Open-field behavioral test

The open-field behavioral test was performed similar to that described previously.^6^ Three months after LIB exposure, each mouse was placed in a square open-field arena (40 × 40 cm, with a 30-cm wall) (RRID:SCR_004074; Noldus Information Technology, Leesburg, VA, USA) with a virtually partitioned 10 × 10 cm center zone (Fig. 1B). Mice were tracked for 10 min using the EthoVision software (RRID:SCR_000441; EthoVision HTP 2.1.2.0, based on EthoVision XT 4.1, Noldus Information Technology, Wageningen, The Netherlands). Arenas were cleaned with 20% ethanol and air-dried before each test. Total distance traveled, time moving, center zone duration, and center zone frequency were measured.

### Social interaction test

Mice were subjected to the social interaction test in the same arena used for the open-field behavioral test (Fig. 1C) and tracked both with the EthoVision software and manually by investigators at a later period. Mice were assigned to three types of interaction pairs: (1) LIB-exposed mouse + LIB-exposed mouse (B + B), (2) sham control + LIB-exposed mouse (S + B), or (3) sham control + sham control (S + S). During the beginning of testing, two mice housed in different cages were placed at opposite ends of the arena, facing the wall. Both unfamiliar mice were considered “test” mice. Mice were then allowed to interact freely and unconfined for 15 min. The duration of no-body contact, representing intervals where the two interaction mice were not in contact, was analyzed with the EthoVision for the first 5 min of testing.

To investigate individual mouse differences, two investigators performed manual analyses. The mouse was defined as an initiator upon demonstrating an intentional approach toward the other subject, ending in nose-on contact/sniffing. If both subjects equally approached each other, as in instances where both mice came into contact incidentally during arena exploration, they were both defined as the initiator. For another initiation to be counted, the mouse had to disengage by turning their heads away from one another or walking away. All instances where the subject mouse initiated, including instances where both mice initiated equally, were calculated.

### The Erasmus Ladder test

The Erasmus Ladder test measures motor coordination-dependent learning, gait adaptation, and associative learning.^27^ This apparatus consists of a horizontal ladder with 37 pairs of rungs in alternating high and low positions, each equipped with touch sensors (Fig. 1D). As the mouse walks on the ladder, information is sent to the program. A variety of step types are measured: short steps, long steps, jumps, missteps, and backsteps. Stepping from one high rung to the following high rung is considered a short step, one high rung to two rungs down is a long step, one high rung to more than two high rungs down is a jump, one high rung to a low rung is a misstep, and taking a step backward to a high or low step is a backward step.^28^ Motor coordination is assessed by missteps, while gait adaptation entails the transition from short to long steps.^29^

The default protocol for this experiment consisted of 42 trials per session. One session was performed per day for eight consecutive days. The first four sessions were training sessions entailing undisturbed trials, while the last four sessions were challenge sessions that varied in trial type. Regardless of session, resting period in the starting box of 15 ±-5 sec preceded each trial. Each mouse had to complete the full duration of the resting period for the trial to begin. If the mouse left prematurely, an air cue from the opposite side of the ladder was turned on to encourage the mouse to remain in the starting box. Once the trial started, a light cue was turned on in the starting box to signal the mouse to exit. If the mouse did not exit the box, an air cue was turned on in the starting box to influence the mouse to exit. As the mouse began to walk on the ladder, a tail wind generated by pressurized air outlets was followed until the mouse reached the ending goal/resting box. If the mouse paused for longer periods to sit on the ladder rungs, an investigator would encourage the mouse to continue walking by gently pressing the back of the mouse.

Trial types in challenge sessions 5–8 included a trial with an unconditioned stimulus only (US-only, stimulus: perturbation where obstacle rung is activated), a conditioned stimulus only (CS-only, stimulus: a high-pitch tone), or paired (CS+US, a tone precedes perturbation by 250 ms). Program detection enabled the investigation of mouse behavior pre- and post-perturbation, gauging information on step patterns and durations. For challenge sessions, absolute learning was calculated as the amount of time taken to execute a step during pre- and post-perturbation.^30^

### Data and statistical analyses

Data were analyzed using Prism software (RRID:SCR_002798; GraphPad Software, La Jolla, CA). Data are presented as mean values ± Standard Error of the Mean (SEM). For the open-field behavior test, the unpaired one-tailed Student’s *t*-test was used for group comparisons of distance moved, time moving, duration in center zone, and center zone frequency. Percentage (%) differences presented for open-field parameters were calculated using the following equation: (blast value − sham value)/sham value. For the social interaction test, a one-way Analysis of Variance (ANOVA) was used to analyze pair-type data and an unpaired one-tailed Student’s *t*-test was used to analyze the initiation number and percentage. The remaining parameters representing multiple time points were analyzed by repeated-measures two-way ANOVA with the Geisser–Greenhouse correction, followed by the multiple-comparisons Bonferroni post-test. A *p* value of < 0.05 was considered statistically significant.

## Results

### Open-field behavioral test in the chronic phase post-LIB exposure

**FIG. 2.**
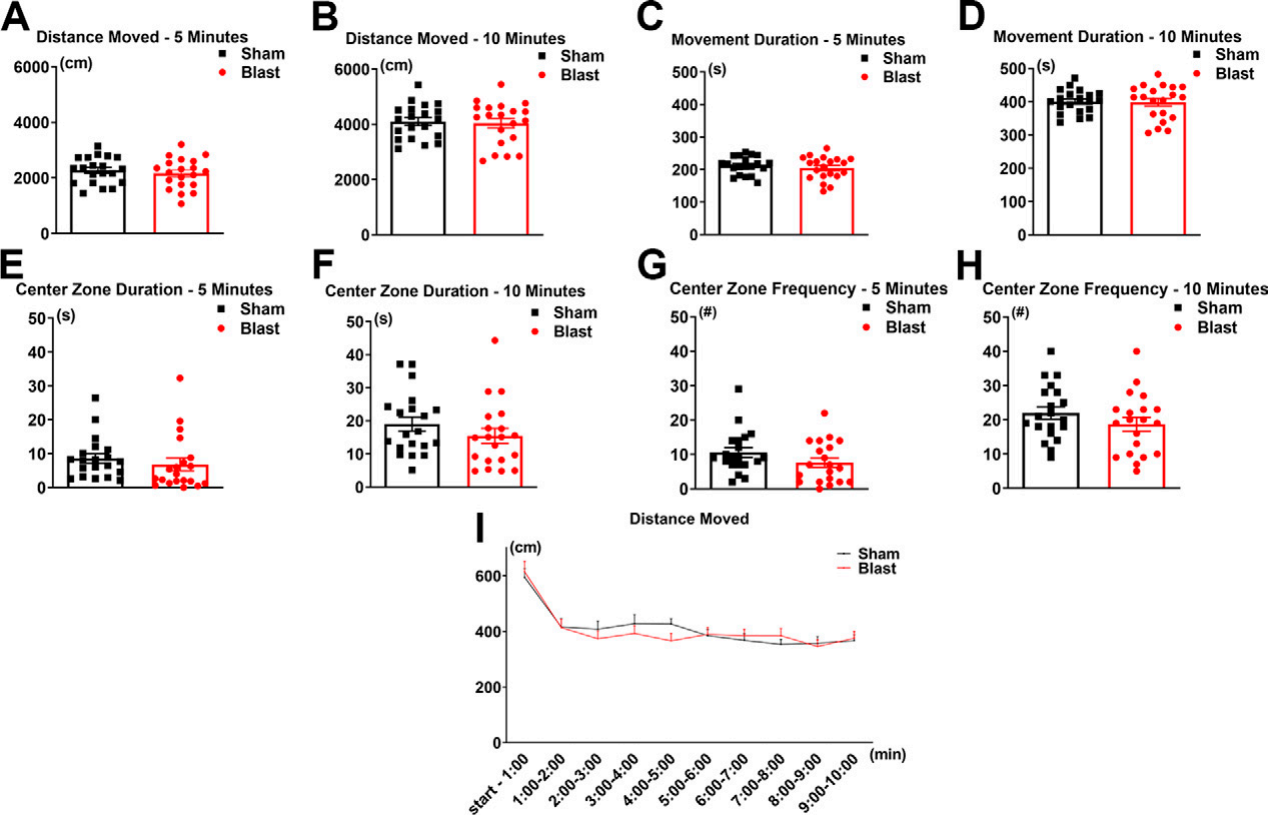
Open-field test assessment. Total distance moved **(A, B)**, movement duration **(C, D)**, center zone duration **(E, F)**, and center zone frequency **(G, H)** are displayed for a total of 5 and 10 min for LIB-exposed mice and sham controls. Distance moved for LIB-exposed mice and sham controls is displayed in 1-min time bins for 10 min **(I)**. *p* > 0.05 by one-tailed, unpaired Student’s *t*-test **(A–H)**. Data are expressed as mean ± SEM. *n* = 20 sham and *n* = 20 LIB-exposed mice. LIB, low-intensity blast.

In the current study, the long-term effects of LIB exposure on behavior were evaluated using the open-field test (OFT) analyzed within a 10-min duration for temporal changes at 3 months post-injury. Results revealed that LIB-exposed mice traveled 5% less total distance in the first 5-min segment of testing and 2% less for the entire 10-min testing duration as compared with sham controls, although significance was not reached (*p* = 0.2472 for 5 min; *p* = 0.3920 for 10 min; Fig. 2A, B). Movement duration was reduced by 4% for 5 min and 0% for 10 min for the LIB-exposed mice, with no significant differences (*p* = 0.2251 for 5 min; *p* = 0.4462 for 10 min; Fig. 2C, D). Center zone duration was reduced by 21% for the LIB-exposed mice for 5 min and 19% for the entire testing duration, with no significant differences (*p* = 0.2216 for 5 min; *p* = 0.1257 for 10 min; Fig. 2E, F). For center zone frequency, LIB-exposed mice displayed a 28% reduction for 5 min and a 15% reduction for 10 min, with no significant differences (*p* = 0.0650 for 5 min; *p* = 0.1151 for 10 min; Fig. 2G, H). To further view detailed temporal changes in distance moved across shorter segments of time, data were analyzed in 1-min time bins and no significant effect of LIB was found (*p* = 0.7840; Fig. 2I). Overall, these results showed small trends but non-significant differences in anxiety-like behaviors.

### Behavioral test on social interaction in the chronic phase post-LIB exposure

**FIG. 3.**
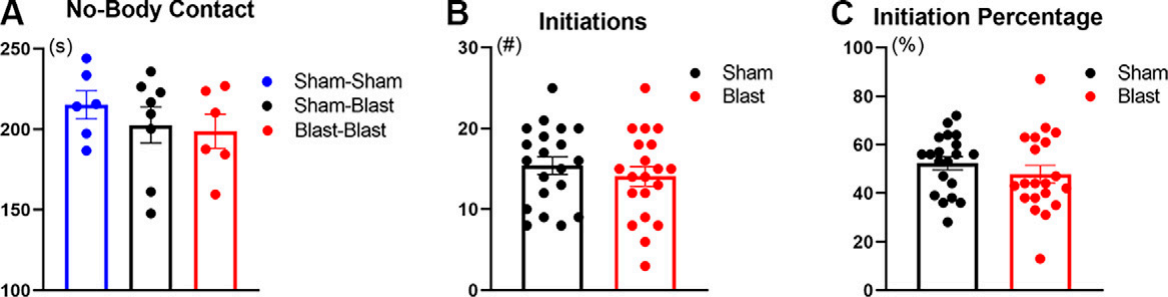
Social interaction test assessment. The duration of no-body contact for blast–blast, blast–sham, and sham–sham interactions are compared for the initial 5 min of testing **(A)**. The number of initiations made by sham and blast were compared for the initial 5 min of testing **(B)**. Initiation percentage **(C)**, reflecting the percentage of total pair interactions initiated and initiation number were assessed for the initial 5 min between LIB-exposed mice and sham controls. *p* > 0.05 by one-tailed, unpaired Student’s *t*-test. Data are expressed as mean ± SEM. *n* = 20 in sham and *n* = 20 LIB-exposed mice. LIB, low-intensity blast.

Sociability impairments are important long-term complications in TBI patients and animal models, albeit few such studies have focused on LIB-induced mTBI.^31–33^ Therefore, in the current study, we used the open-field arena to test social interaction in mice 3 months after LIB injury. Two unfamiliar mice were simultaneously placed in the open-field arena, facing a corner at start and enabled free access to interact for 15 min. A one-way ANOVA revealed no significance (*p* = 0.5588) between pair types for the 5-min duration of no-body contact (Fig. 3A). For manual analysis, initiations were analyzed in number and percentages were calculated to determine how often mice initiated compared with their partner. No significant differences were found (*p* = 0.2071 for initiation number and *p* = 0.1633 for initiation percentage; Fig. 3B, C). Interestingly, 13/20 LIB-exposed mice (65%) and 7/20 sham mice (35%) initiated less than half (<50%) of the interactions made in their pair. Although the assessment did not reveal significant differences, these data suggest a tendency for reduced social motivation or “preference” in the presence of “novel conspecifics”^34^ in the LIB-exposed mice.

### Erasmus Ladder performance in the chronic phase post-LIB exposure

**FIG. 4.**
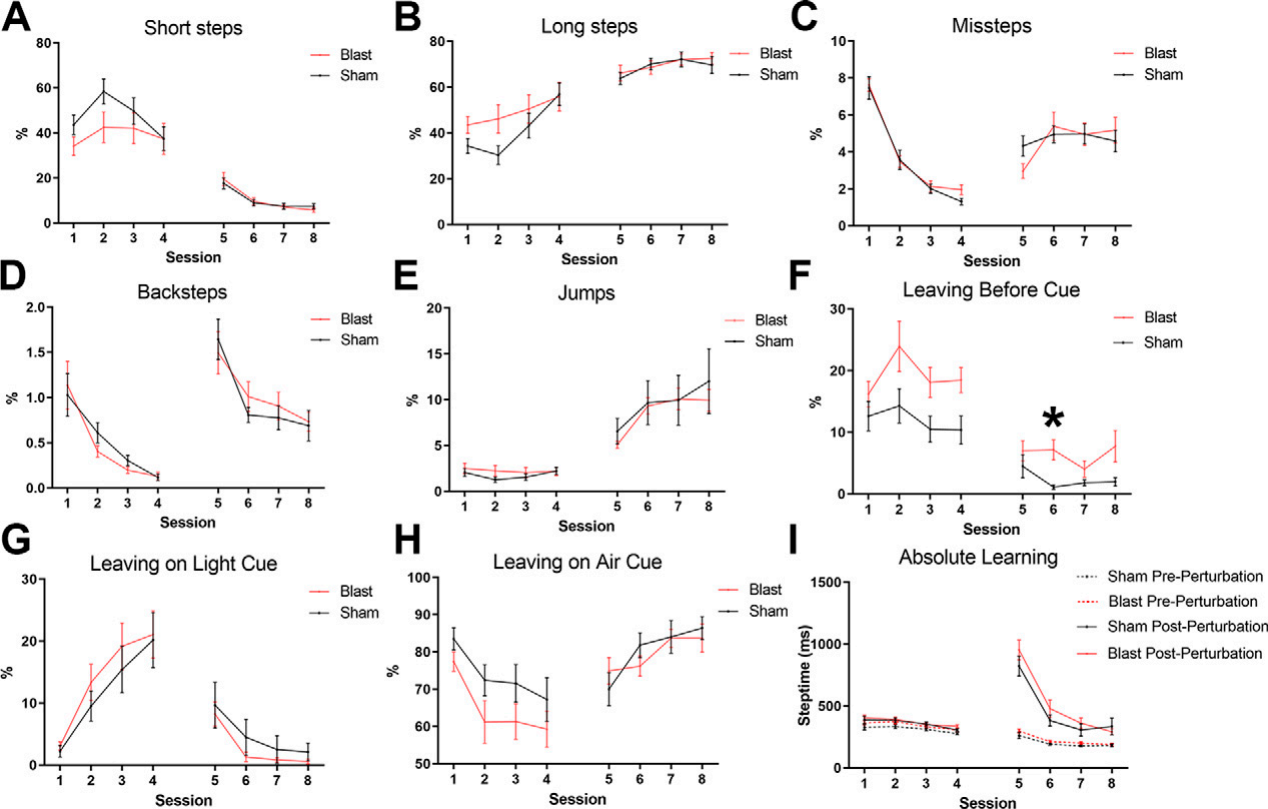
The Erasmus Ladder test assessment. Percentage of short steps **(A)**, long steps **(B)**, missteps **(C)**, backsteps **(D)**, and jumps **(E)** in unchallenged (sessions 1–4) and challenged sessions (sessions 5–8). Percentages for leaving before cue **(F)**, leaving on light cue **(G)**, and leaving on air cue **(H)** for training and challenge sessions. Absolute learning was calculated as total milliseconds to complete step post-perturbation (unconditioned stimulus) in challenged sessions and calculated for comparison without perturbation in unchallenged sessions **(I)**. Data are expressed as mean ± SEM. *p < 0.05 using Bonferroni post-test and Geisser–Greenhouse correction. For panels **A–H**, *n* = 17 sham mice and *n* = 20 LIB-exposed mice. For panel **I**, *n* = 16 sham mice and *n* = 20 LIB-exposed mice. LIB, low-intensity blast.

The Erasmus Ladder is an advanced, automated, and multifaceted behavioral assessment system used in neuroscientific studies.^28,30^ Repeated-measures ANOVA was performed to determine the effects of LIB on step types, including short steps, long steps, missteps, backsteps, and jumps (Fig. 4A–E). We first sought to analyze the effects of LIB on motor functions of the Erasmus Ladder test. We observed that LIB had no significant effect on any step type: short steps (*p* = 0.3771), long steps (*p* = 0.3457), missteps (*p* = 0.8961), backsteps (*p* = 0.9722), and jumps (*p* = 0.8570; Fig. 4A–E). These findings indicated that motor function was unaltered following LIB injury.

Next, we examined activities on associative learning and mouse responses to box departure cues, including leaving the starting box before cue, leaving on light cue, or leaving on air cue (Fig. 4F–H) as well as absolute learning (Fig. 4I). LIB had a significant effect on instances of leaving before cue (*p* = 0.0062). A Bonferroni post-test revealed a significant difference for session 6 (*p* = 0.0127; Fig. 4F). LIB had no significant effect on the percentage of instances for leaving on light cue (*p* = 0.9450) or air cue (*p* = 0.1863; Fig. 4G–H). LIB had a significant effect on pre-perturbation step times (*p* = 0.0189). Associative learning was evaluated by US (perturbation)-response behavior, or post-perturbation step times, with no effect of LIB (*p* = 0.2389; Fig. 4I). Overall, the Erasmus Ladder revealed intact motor functioning and ladder-related associative learning; however, the presence of escape behaviors suggested anxiety-related activity following LIB exposure.

## Discussion

Individuals with blast-induced mTBI can develop long-lasting physical and mental deficits that degrade their quality of life.^35^ Nevertheless, efforts to identify these long-lasting neurobehavioral abnormalities in LIB animal models have been challenging, which may be partly due to the need for multi-domain and advanced apparatus capable of detecting subtle changes. In this study, we implemented neurobehavioral evaluations with the “Missouri Blast” mouse model using the open-field, social interaction, and Erasmus Ladder tests 3 months after LIB exposure.

Using the OFT, our group previously reported reduced motor activity and anxiety-related behaviors in the acute phase of post-LIB injury, as evidenced by significantly less distance traveled at 3 days post-injury and significantly less center zone distance traveled at 6 days post-injury compared with sham controls.^6^ In previous studies, LIB exposures had shown to induce primary brain injuries in subcellular ultrastructures, such as impairments of mitochondria, asymmetrical synapses, myelinated axons, microvessels, and tight junction structures, as well as altered molecular subnetworks.^5,6,23,24,36^ In the current study, assessment using the OFT to mice at 3 months after LIB exposure revealed no significant differences in distance moved or movement duration at 5 or 10 min between LIB-exposed mice and sham controls. However, despite not attaining statistical significance, there were apparent trends for decreased center duration and frequency for both 5 and 10 min for the LIB-exposed mice (Fig. 2E–H). These results indicate that the conventional OFT may have limitations in detecting subtle long-term anxiety-related behaviors following LIB injury.

Our previous studies using the automated home-cage monitoring (aHCM) platform in the spontaneous activity and LightSpot tests^22^ showed significant differences between LIB-exposed mice and sham controls, with multiple parameters relevant to anxiety-like behaviors. These observations suggest that LIB-exposed mice may hold stable perceptions of environmental stimuli as a threat, as compared with sham controls. This type of performance aligns with trait anxiety in humans, characterized by a tendency to respond negatively (e.g., worry or concern) to non-threatening situations.^37,38^

Sociability impairments are relevant long-term complications in TBI patients, although studies focusing on LIB-induced mTBI have not been extensive.^31–33^ Despite of different ways to assess social interaction, such as the three-chamber protocol as described by Bicks et al. 2020,^39^ other studies such as the one by Jabarin et al. 2022^40^ also indicated advantage using the open-field arena for assessment of social interaction. Using the open-field arena, LIB-exposed mice displayed a trend for lower initiation of social interaction as compared with their interaction partner at 3 months post-injury (Fig. 3B, C). In fact, 65% of LIB-exposed mice initiated less than 50% of the interactions with their partner. These findings, despite lacking statistical significance, suggest a focus for future studies to examine and confirm whether LIB-exposed mice may develop lower sociability, reduced social motivation, or “preference” in the presence of “novel conspecifics.”^34,41^ This feature is consistent with clinical conditions of impaired neurobehavioral initiation and motivation in patients with brain injury.^42^ As multiple cortical and subcortical regions are involved,^42^ future studies with region-specific Functional magnetic resonance imaging (fMRI) may be useful for post-blast veterans with motivational disorders.

Using the Erasmus Ladder behavioral test, LIB-exposed mice displayed a significant increase in percentage of escaping the resting box before cues, suggesting a heightened sensitivity or aversiveness to the box-relevant cues. This change may reflect behaviors analogous to anxiety sensitivity, a state of anxiety due to the anticipation of aversive stimuli that motivates escape behaviors.^43,44^ The preference for long steps among LIB-exposed mice, indicating accelerated gait adaption,^29^ may also support anxiety-related behaviors. Taken together, these results suggest that subtle anxiety-like behaviors in the chronic phase post-LIB exposure can be assessed with multifaceted behavioral tests, consistent with our recently published study with the aHCM system, which revealed distinct spontaneous and affective behaviors in LIB-exposed mice 3 months post-injury in both natural and challenged conditions.^22,45,46^ However, missteps on the Erasmus Ladder test and responses to the US (perturbation) did not differ between LIB-exposed mice and sham controls, this result indicated that deficits of motor coordination and association learning may not be predominant long-term consequences of LIB exposure (Fig. 4A–I). Nevertheless, LIB-induced deficits in discrimination learning and cognitive flexibility were observed by the aHCM CognitionWall test.^23^

In many instances, individuals with trait anxiety also demonstrated decrease in social competitiveness.^47^ These behavioral changes, including the multifaceted anxiety-like behaviors and decreased motivation observed at 3 months post-LIB, may be linked to altered proteomes in subnetworks and subcellular neuropathology previously observed during acute and subacute phases after LIB exposure.^6,24^ Furthermore, another potential mechanism may involve the disruption of the amygdala and the hypothalamic-pituitary-adrenal axis, which is linked to impaired stress response following TBI.^48–50^ Nevertheless, the ability to identify whether LIB (as low as 46.6 kPa) can induce amygdala damage by applying highly sensitive methods, such as ultrastructural assessments and proteomic/transcriptomic analyses, will be of importance for further understanding aberrant behavioral functions after LIB.

## Conclusions

In summary, with the application of multifaceted behavioral assessments, our study revealed long-lasting anxiety-like outcomes in mice 3 months following single LIB exposure. These changes in behavioral outcomes are vital in the context of our previous neurophysiological and pathological findings. Thus, the “Missouri Blast” LIB mouse model offers a realistic translational platform to investigate the effects of military-relevant blast exposure not only on molecular networks and targets but also behavioral deficits associated with progressive neurodegeneration and dementia in the affected population. With the current platform, future LIB studies from bench to bedside and back may increase our understanding of the pathophysiological process and provide insights for therapeutic developments.

## Abbreviations Used

aHCM: Automated home-cage monitoring
CS: Conditioned stimulus
fMRI: Functional magnetic resonance imaging
LIB: Low-intensity blast
mTBI: Mild traumatic brain injury
OFT: Open field test
US: Unconditioned stimulus
